# Diagnostic performance of non-invasive liver fibrosis scores in patients with early-intermediate hepatocellular carcinoma

**DOI:** 10.1007/s00432-024-05708-3

**Published:** 2024-04-11

**Authors:** Christopher Schrecker, Falko Schulze, Jörg Trojan, Wolf Otto Bechstein, Stefan Zeuzem, Christine Koch

**Affiliations:** 1 Department of Medicine, Goethe University Frankfurt, University Hospital, Frankfurt am Main, Germany; 2 Dr. Senckenberg Institute of Pathology, Goethe University Frankfurt, University Hospital, Frankfurt am Main, Germany; 3 Department of Surgery, Goethe University Frankfurt, University Hospital, Frankfurt am Main, Germany; 4 Frankfurt Institute of Clinical Cancer Research, Krankenhaus Nordwest, Frankfurt am Main, Germany

**Keywords:** HCC, Fibrosis, Cirrhosis, FIB-4, APRI

## Abstract

**Purpose:**

Hepatocellular carcinoma (HCC) arises in individuals with underlying liver disease. Diagnosing the degree of hepatic fibrosis helps to determine the severity of the underlying liver disease and may influence therapeutic decisions in HCC patients. Non-invasive fibrosis scores can be used to estimate the degree of fibrosis in liver disease patients, but most of these scores were developed in patients with viral hepatitis and without HCC. This study explored the ability of the Fibrosis-4 Index (FIB-4), the AST/Platelet Ratio Index (APRI), and the AST/ALT ratio to diagnose or exclude advanced fibrosis (METAVIR F3/4 versus F0-2) in patients with early-intermediate, potentially resectable HCC.

**Methods:**

We retrospectively reviewed 119 patients who underwent hepatic resection for HCC at a tertiary centre (2007–2019), 75 of whom had advanced fibrosis (prevalence 63%). Histological assessment of the surgical liver specimen was used as a reference standard for the degree of fibrosis.

**Results:**

Overall diagnostic performance was highest for the FIB-4 Index, with an area under the receiver operating characteristic curve (AUROC) of 0.82, compared with 0.78 for APRI, and 0.56 for the AST/ALT ratio. Using established cut-off values, FIB-4 achieved a 90% positive predictive value at the higher cut-off (3.25) and a 90% negative predictive value at the lower cut-off (1.45).

**Conclusion:**

The FIB-4 Index could reliably diagnose or exclude advanced fibrosis in patients with early-intermediate HCC, and may thus have a role in guiding therapeutic decisions in these patients.

## Introduction

Hepatocellular carcinoma (HCC) is a leading cause of cancer-related morbidity and mortality (Bray et al. 2018). HCC typically affects patients with chronic viral hepatitis, alcoholic liver disease, or metabolic dysfunction-associated steatohepatitis (Villanueva 2019). Treatment of HCC is guided by tumour size and spread, as well as the severity of the underlying liver disease (Galle et al. 2018; Heimbach et al. 2018). For example, patients with early-intermediate HCC and advanced liver disease may be better candidates for locoregional therapies than surgical resection, because poor hepatic reserve increases perioperative risk and mortality (Llovet et al. 1999). Liver disease severity must therefore be accurately determined in patients with early-intermediate, potentially resectable HCC.

Hepatic fibrosis is a key determinant of liver disease severity, alongside liver synthetic function and the presence of portal hypertension (Ge and Runyon 2016). In patients with HCC, the extent of hepatic fibrosis can influence overall prognosis, the likelihood of complications, and the choice and feasibility of different treatment options (Allaire et al. 2020; Wang et al. 2013). For example, perioperative risk, including the likelihood of posthepatectomy liver failure, is greater in patients with histological evidence of cirrhosis (Farges et al. 1999; Liang et al. 2022). Moreover, advanced fibrosis increases the risk of late recurrence following resection, likely due to an increased rate of de novo tumour formation within the fibrotic liver (Ko et al. 2002; Taura et al. 2007). Diagnosing advanced fibrosis in patients with potentially resectable HCC may therefore tip the balance in favour of locoregional therapies rather than surgical resection, given the increased perioperative risk on the one hand, and the relatively low chance of long-term, recurrence-free survival on the other hand.

Liver biopsy is the gold standard for diagnosing hepatic fibrosis and is also the modality of choice for diagnosing HCC (Galle et al. 2018). Hence, when patients with suspected HCC undergo liver biopsy, histological assessment of the adjacent non-tumour tissue can aid in determining the degree of liver fibrosis. However, histological assessment of fibrosis may be inaccurate or inconclusive, even in patients without HCC (Tapper and Lok 2017). Moreover, liver biopsy is not required when HCCs can be diagnosed by contrast-enhanced imaging (Galle et al. 2018; Heimbach et al. 2018). Thus, alternative strategies are needed to assess the degree of hepatic fibrosis in patients with early-intermediate HCC.

In the present study, we explored whether non-invasive fibrosis scores could be used instead of liver biopsy to diagnose hepatic fibrosis in patients with early-intermediate HCC. Most fibrosis scores were established and validated in patients with viral hepatitis and other forms of chronic liver disease (Lin et al. 2011; McPherson et al. 2010; Shaheen and Myers 2007; Sheth et al. 1998; Sterling et al. 2006; Wai et al. 2003), but have not been formally tested in HCC. We reasoned that HCCs might impair the diagnostic accuracy of fibrosis scores, by affecting transaminase levels and platelet counts which are used to calculate the scores. Our study investigated the performance of the Fibrosis-4 Index (FIB-4), the AST/Platelet Ratio Index (APRI) and the AST/ALT ratio in diagnosing or excluding advanced hepatic fibrosis (METAVIR F3/4 versus F0-2) in cases of early-intermediate HCC.

## Materials and methods

### Study design

In this retrospective cross-sectional study, we reviewed clinical and laboratory data from 119 patients who underwent hepatic resection for HCC (February 2007–May 2019) at the University Hospital Frankfurt, a German referral centre for liver diseases. Inclusion criteria were biopsy-proven HCC (BCLC stages 0/A or B), recommendation of surgery by a multidisciplinary tumour board, and hepatic resection with curative intent. The patients in this study represent a subset of a previously published cohort of 128 patients (Schrecker et al. 2022), from which we excluded 9 patients due to incomplete laboratory values which are required for calculation of the liver fibrosis scores. The study was approved by the institutional review board of the University Hospital Frankfurt (reference number SGI-4-2019).

### Definitions

Tumours were staged according to the Barcelona Clinic Liver Cancer (BCLC) system (Llovet et al. 1999). BCLC stage A is defined as early HCC and BCLC stage B as intermediate HCC. Solitary tumours without macrovascular invasion or extrahepatic spread were classified as BCLC stage A irrespective of their size (Galle et al. 2018). Child–Pugh class was determined as detailed in the original publication (Child and Turcotte 1964). The histological degree of fibrosis was graded according to the METAVIR scoring system in the surgical liver specimen (F3/F4 for presence of advanced fibrosis/cirrhosis, versus F0-F2 for absence of advanced fibrosis) (Bedossa 1994; Bedossa and Poynard 1996). Fibrosis-4 Index (FIB-4) and APRI were calculated as follows: FIB-4 = (Age × AST [U/L])/(platelets [/nL] × √ALT [U/L]) (Sterling et al. 2006); APRI = 100 × (AST [U/L]/AST upper limit of normal [40 U/L])/platelets [/nL] (Wai et al. 2003). For calculation of the AST/ALT ratio, AST and ALT are expressed in U/L. All laboratory parameters used to calculate the fibrosis scores were measured preoperatively. Higher FIB-4, APRI and AST/ALT values indicate more advanced hepatic fibrosis.

### Statistical analysis

Data are expressed as mean ± SEM. Statistical analyses were carried out in GraphPad Prism Version 9. Comparisons were made using unpaired *t* tests for continuous variables and Chi-square tests to compare the frequency distribution between categorical variables. Fisher’s exact test was used instead of the Chi-square test for contingency tables with cell counts below 5. Results with a *p*-value < 0.05 were considered statistically significant. Area under the receiver operating characteristic curve (AUROC) was used as a global measure of test performance for the fibrosis scores. Other test performance metrics (sensitivity, specificity, positive and negative predictive values) were calculated as outlined in (Trevethan 2017).

## Results

The study cohort comprised 119 patients who underwent hepatic resection for HCC. Amongst these, 83 individuals were diagnosed with BCLC stage 0/A tumours, whilst 36 had BCLC stage B tumours. Histological assessment of liver specimens revealed that 75 patients had F3/4 fibrosis (prevalence 63%), whereas 44 exhibited no or mild fibrosis (F0-2). Hepatic function was preserved in all study participants, with 117 classified as Child–Pugh A and two as Child–Pugh B7. Baseline characteristics for the full cohort, as well as separately for patients with F0-2 and F3/4 fibrosis, are presented in Table 1.

**Table 1:** Comparison of baseline characteristics between the 44 patients with no or mild fibrosis (METAVIR F0-2) and the 75 patients with advanced fibrosis (METAVIR F3/4)

Variables	Full cohort (119)	F0-2 (44)	F3/4 (75)	*p*-Value^1^
Age (years)	64 ± 1^2^	63 ± 2	64 ± 1	0.36^a^
Gender (male)	88 (74%)	35 (80%)	53 (71%)	0.29^b^
Liver disease aetiology				
HBV/HCV	55 (46%)	16 (36%)	39 (52%)	
ASH/NASH	46 (39%)	18 (41%)	28 (37%)	0.12^b^
Other	18 (15%)	10 (23%)	8 (11%)	
BCLC stage				
0/A	83 (70%)	28 (64%)	55 (73%)	0.27^b^
B	36 (30%)	16 (36%)	20 (27%)	
Child–Pugh class				
A	117 (98%)	44 (100%)	73 (97%)	0.53^c^
B7	2 (2%)	0 (0%)	2 (3%)	
AST (U/L)	60 ± 4	55 ± 8	63 ± 5	0.40^a^
ALT (U/L)	59 ± 7	63 ± 17	57 ± 6	0.69^a^
Platelet count (/nL)	195 ± 8	260 ± 13	157 ± 8	< 0.001^a^
FIB-4	3.58 ± 0.28	2.04 ± 0.22	4.48 ± 0.38	< 0.001^a^
APRI	1.04 ± 0.11	0.53 ± 0.06	1.34 ± 0.17	< 0.001^a^
AST/ALT ratio	1.38 ± 0.10	1.36 ± 0.17	1.40 ± 0.12	0.83^a^

*APRI* AST/Platelet Ratio Index, *ASH/NASH* alcoholic/non-alcoholic steatohepatitis, *AST* aspartate transaminase, *ALT* alanine transaminase, *BCLC* Barcelona Clinic Liver Cancer, *FIB-4* Fibrosis-4 Index, *HBV* hepatitis B virus, *HCV* hepatitis C virus

^1^*p*-values were calculated by ^a^unpaired *t* test, ^b^Chi-square test, or ^c^Fisher’s exact test

^2^Continuous data are expressed as mean ± SEM

**Fig. 1:**
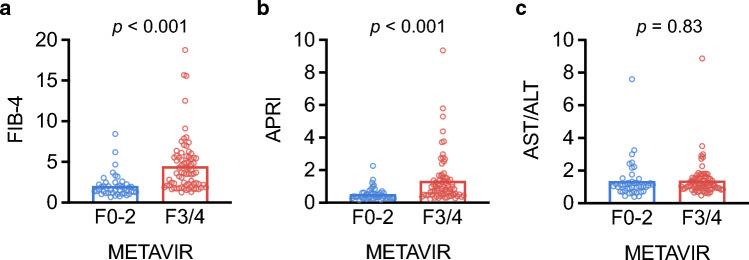
Correlation of non-invasive fibrosis scores and histological degree of liver fibrosis. **a**–**c** Results of unpaired *t* test analyses showing the association of METAVIR score with **a** the Fibrosis-4 Index (FIB-4), **b** the AST/Platelet Ratio Index (APRI) and (**c**) the AST/ALT ratio, in 119 patients with early-intermediate HCC. Results with a *p*-value < 0.05 were considered statistically significant

We first compared baseline characteristics and fibrosis score values between patients with F0‑2 and F3/4 fibrosis (Table 1 and Fig. 1). On average, patients with F3/4 fibrosis had higher FIB‑4 and APRI scores, but mean AST/ALT ratios did not differ between the groups. In addition, patients with F3/4 fibrosis had lower platelet counts, which is a classic hallmark of advanced liver disease. Other baseline characteristics including age, liver disease aetiology and BCLC stage were comparable between patients with F0‑2 and F3/4 fibrosis.

**Fig. 2:**
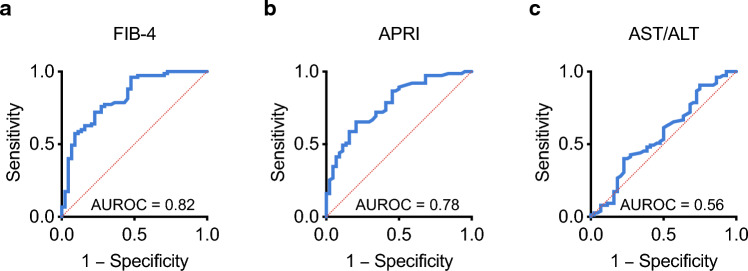
Area under the receiver operating characteristic curve (AUROC) values for the different liver fibrosis scores. **a**–**c** Results of AUROC analyses for **a** the Fibrosis-4 Index (FIB-4), **b** the AST/Platelet Ratio Index (APRI) and **c** the AST/ALT ratio, in 119 patients with early-intermediate HCC

Next, we assessed the diagnostic performance of the different fibrosis scores (Table 2 and Fig. 2), using the area under the receiver operating characteristic curve (AUROC) as a global measure of test performance. Table 2 also summarises sensitivity, specificity, positive predictive value (PPV) and negative predictive value (NPV) for the different scores. Overall performance was good for FIB-4 and APRI, with AUROC values of 0.82 and 0.78, respectively. Using established cut-off values (Sterling et al. 2006), FIB-4 achieved a 90% PPV at the higher cut-off (3.25) and a 90% NPV at the lower cut-off (1.45). PPV was equally high for APRI (89%) at the previously reported cut-off of 1.0 (Lin et al. 2011), but NPV was comparatively low (58%) even at a lower cut-off (0.5). Unlike FIB-4 and APRI, the AST/ALT ratio performed poorly overall (AUROC 0.56).

**Table 2:** Performance of the different fibrosis scores in 119 patients with early-intermediate HCC

Test	AUROC (95% CI)	Cut-off	Sens (%)	Spec (%)	PPV (%)	NPV (%)
FIB-4	0.82 (0.74–0.90)	1.45	97	41	74	90
		3.25	57	89	90	55
APRI	0.78 (0.70–0.86)	0.5	75	59	76	58
		1.0	44	91	89	49
		1.5	25	98	95	43
AST/ALT	0.56 (0.46–0.67)	0.8	87	27	67	55
		1.0	67	36	64	39

*AUROC* area under the receiver operating characteristic curve, *APRI* AST/Platelet Ratio Index, *CI* confidence interval, *FIB-4* Fibrosis-4 Index, *NPV* negative predictive value, *PPV* positive predictive value, *Sens* sensitivity, *Spec* specificity

Given its excellent performance metrics, we focussed our analysis on the FIB-4 Index (Table 3). Using 1.45 as the lower and 3.25 as the upper cut-off point, FIB-4 classified 20/119 patients (17%) as F0-2, 48/119 (40%) as F3/4, and 51/119 (43%) as indeterminate. Out of the 68 individuals who were classified as either F0-2 or F3/4, only 7 were misclassified, owing to the low false positive and false negative rates (both 10%). Taken together, we show that the FIB-4 Index can reliably diagnose or exclude advanced fibrosis in a majority of patients with early-intermediate HCC. However, alternative assessment of liver fibrosis will be required in patients with an indeterminate FIB-4 score.

**Table 3:** Proportion of patients who were correctly classified using the FIB-4 Index

Fibrosis classification	FIB-4 score	No. of patients	Correctly classified	Incorrectly classified
None/mild (F0-2)	< 1.45	20/119 (17%)	18/20 (90%)	2/20 (10%)^1^
Indeterminate	≥ 1.45 and < 3.25	51/119 (43%)	NA	NA
Advanced (F3/4)	≥ 3.25	48/119 (40%)	43/48 (90%)	5/48 (10%)^2^
All patients	NA	119/119 (100%)	61/68 (90%)	7/68 (10%)

^1^False negatives

^2^False positives

## Discussion

Our study explored whether non-invasive fibrosis scores could reliably discriminate between no or mild fibrosis (METAVIR F0-2) and advanced fibrosis (METAVIR F3/4) in patients with early-intermediate HCC. Using previously established cut-off values (Lin et al. 2011; Sheth et al. 1998; Sterling et al. 2006), we found that the FIB‑4 Index had high positive and negative predictive values (PPV/NPV) for diagnosing or excluding advanced fibrosis, APRI had high PPV but low NPV, and the AST/ALT ratio had poor overall performance (Table 2). Our initial hypothesis was that HCCs might compromise the diagnostic accuracy of fibrosis scores, by affecting transaminase levels and platelet counts which are used to calculate the scores. Our findings, however, do not support this assumption and nominate the FIB-4 Index as a reliable method to determine the degree of hepatic fibrosis in patients with early-intermediate HCC.

Patients with suspected HCC usually undergo liver biopsy, and histological assessment of the adjacent non-tumour tissue can be used to determine the degree of hepatic fibrosis. However, interpretation of biopsies with respect to fibrosis is subjective, and may be inaccurate when biopsy specimens are not large enough, or when liver disease does not affect the liver uniformly (Tapper and Lok 2017). Moreover, not all patients with suspected HCC undergo liver biopsy, because HCC can instead be diagnosed by contrast-enhanced imaging (Galle et al. 2018; Heimbach et al. 2018). In clinical situations where biopsy data is unavailable, inconclusive, or inconsistent with the overall clinical picture, the FIB‑4 Index may therefore be a reliable alternative for diagnosing liver fibrosis.

The excellent diagnostic performance of the FIB-4 Index was unexpected because transaminase levels and platelet counts, both of which are used to calculate FIB-4, may be affected by HCCs. For example, previous studies showed that HCCs can increase the AST/ALT ratio (von Felden et al. 2020) and may cause a paraneoplastic increase in platelet counts (Hwang et al. 2004). In our cohort, however, HCCs did not negatively affect the accuracy of the FIB-4 Index. In fact, the diagnostic performance of FIB-4 in our cohort (Table 2) was comparable, if not superior, to its performance in the original FIB-4 study (AUROC 0.77, PPV 65% at 3.25 cut-off, NPV 90% at 1.45 cut-off) (Sterling et al. 2006), and to that of APRI in a meta-analysis of 13 APRI studies (AUROC 0.80, PPV 40% and NPV 81% both at 1.0 cut-off) (Lin et al. 2011).

Based on the high NPVs and the relatively low PPVs in these prior studies, FIB-4 and APRI are typically used to exclude but not diagnose advanced fibrosis in patients with chronic liver disease (and without HCC). By contrast, FIB-4 had both a high NPV and a high PPV in our cohort, and could thus be used to both exclude and diagnose advanced fibrosis. High disease prevalence is known to increase PPV and lower NPV (H. B. Wong and Lim 2011), so the high prevalence of advanced fibrosis in our cohort (63% compared with 22% in the original FIB-4 study (Sterling et al. 2006) and 28% in the meta-analysis of APRI studies (Lin et al. 2011)) likely contributed to the high PPV of FIB-4 and APRI, and to the low NPV of APRI. The high prevalence of advanced fibrosis is expected in a population of HCC patients, most of whom suffer from underlying chronic liver disease.

The present study is amongst the first to systematically assess the performance of non-invasive fibrosis scores in patients with HCC. In a previous report of 117 patients undergoing hepatectomy for HCC at a Chinese centre, AUROC values for diagnosing or excluding advanced fibrosis were 0.68 for FIB-4 and 0.74 for APRI (Ouyang et al. 2018). The reported AUROC values are slightly lower than in our study, which may reflect differences in patient characteristics between the two cohorts (e.g. all patients in the Chinese cohort had BCLC A tumours, and 82% of patients were HBsAg positive). The positive and negative predictive values reported for FIB-4 (PPV 60%, NPV 85%, cut-off 1.121) and APRI (PPV 66%, NPV 84%, cut-off 0.365) in the Chinese cohort cannot be easily compared to those in our study because of the different cut-off points. To the best of our knowledge, there have been no further studies evaluating the use of non-invasive fibrosis scores in the context of HCC. Beyond their role in diagnosing fibrosis, a growing body of work has shown that non-invasive fibrosis scores can predict outcomes in patients with chronic liver disease, including development of HCC. For example, higher FIB-4 scores have been linked to a greater risk of HCC in chronic HBV infection (Suh et al. 2015a), after HCV eradication (Ioannou et al. 2019), and in patients who consume large quantities of alcohol (Suh et al. 2015b).

In addition to the non-invasive fibrosis scores, ultrasound-based elastography methods represent a well-established alternative to liver biopsy for diagnosing fibrosis (Berzigotti et al. 2021; Lim et al. 2017). However, much like the fibrosis scores, elastography has been tested mostly in patients with chronic liver disease and not in the context of HCC. It remains to be seen whether liver stiffness measurements are reliable in HCC patients, given that elastography may be inaccurate in the setting of acute transaminitis or biliary obstruction (Coco et al. 2007; Millonig et al. 2008; Sagir et al. 2008), both of which can complicate HCC. In addition, liver stiffness has been shown to increase after locoregional therapy of HCC (Abdelaziz et al. 2019). That being said, a small number of studies have indeed demonstrated that elastography has good performance for diagnosing advanced fibrosis or cirrhosis in patients undergoing hepatectomy for HCC (Cescon et al. 2012; J. S. W. Wong et al. 2013), and the calculated cut-off values for liver stiffness were similar to those recommended for patients with viral hepatitis (and without HCC) (Friedrich-Rust et al. 2008).

Lastly, we must acknowledge the limitations of our study. Its retrospective nature restricts our ability to control for potential confounding variables and introduces the possibility of selection bias. Additionally, the relatively small sample size and single-centre design may limit the generalisability of our findings, especially considering the predominantly European ancestry of our patient cohort. In the future, conducting similar investigations in a prospective setting and with a larger, more diverse patient population, will enhance the external validity of our findings.

## Conclusion

The degree of hepatic fibrosis must be accurately determined in patients with early-intermediate HCC, because patients with advanced fibrosis may be better candidates for locoregional therapies than surgical resection. We found that non-invasive assessment of liver fibrosis using the FIB-4 Index is a reliable alternative to liver biopsy for diagnosing or excluding advanced fibrosis, when biopsy data is unavailable or inconclusive. Our study identified the FIB‑4 Index as a simple, inexpensive tool to improve patient stratification and refine therapeutic decisions in early-intermediate HCC.

## Data Availability

The anonymised patient database is available upon request.
